# Basic Fibroblast Growth Factor Stimulates the Proliferation of Bone Marrow Mesenchymal Stem Cells in Giant Panda (*Ailuropoda melanoleuca*)

**DOI:** 10.1371/journal.pone.0137712

**Published:** 2015-09-16

**Authors:** Jun-Jie Wang, Yu-Liang Liu, Yuan-Chao Sun, Wei Ge, Yong-Yong Wang, Paul W. Dyce, Rong Hou, Wei Shen

**Affiliations:** 1 Institute of Reproductive Sciences, Qingdao Agricultural University, Qingdao, Shandong, 266109, China; 2 Key Laboratory of Animal Reproduction and Germplasm Enhancement in Universities of Shandong, College of Animal Science and Technology, Qingdao Agricultural University, Qingdao, Shandong, 266109, China; 3 Chengdu Research Base of Giant Panda Breeding, Chengdu, Sichuan, 610081, China; 4 College of Life Sciences, Qingdao Agricultural University, Qingdao, Shandong, 266109, China; 5 Department of Animal Sciences, Auburn University, Auburn, Alabama, 36849, United States of America; Institute of Zoology, Chinese Academy of Sciences, CHINA

## Abstract

It has been widely known that the giant panda (*Ailuropoda melanoleuca*) is one of the most endangered species in the world. An optimized platform for maintaining the proliferation of giant panda mesenchymal stem cells (MSCs) is very necessary for current giant panda protection strategies. Basic fibroblast growth factor (bFGF), a member of the FGF family, is widely considered as a growth factor and differentiation inducer within the stem cell research field. However, the role of bFGF on promoting the proliferation of MSCs derived from giant panda bone marrow (BM) has not been reported. In this study, we aimed to investigate the role of bFGF on the proliferation of BM-MSCs derived from giant panda. MSCs were cultured for cell proliferation analysis at 24, 48 and 72 hrs following the addition of bFGF. With increasing concentrations of bFGF, cell numbers gradually increased. This was further demonstrated by performing 3-(4,5-dimethyl-2-thiazolyl)-2,5-diphenyl-2-H-tetrazolium bromide (MTT) cell proliferation assay, 5-Bromo-2-deoxyUridine (BrdU) labeling and cell cycle testing. Furthermore, the percentage of MSCs that were OCT4 positive increased slightly following treatment with 5 ng/ml bFGF. Moreover, we demonstrated that the extracellular signal-regulated kinase (ERK) signaling pathway may play an important role in the proliferation of panda MSCs stimulated by bFGF. In conclusion, this study suggests that giant panda BM-MSCs have a high proliferative capacity with the addition of 5 ng/ml bFGF *in vitro*.

## Introduction

Mesenchymal stem cells (MSCs), one kind of multipotent stem cell, have attracted a lot of attention among stem cells researchers, in recent years, due to their ability to self-renew and differentiate into many different cell types. The potential gives them a wide variety of clinical applications [1], including their increasingly being used in tissue engineering and cell-based therapy related fields, ranging from orthopedic to cardiovascular medicine [2]. Noteworthy, the immunosuppressive capacity of MSCs is the biggest advantage within the clinical field, they do not express major histocompatibility class II and can therefore escape immune recognition by the host [1]. Therefore they can survive and form the stromal microenvironment in which the cells can reside and differentiate into different cell types.

In 2013, Liu et al. successfully isolated stem cells from the bone marrow (BM) of the Giant Panda (*Ailuropoda melanoleuca*), and further investigated the proliferation and differentiation potential of these MSCs *in vitro*. It has been widely known that the giant panda is one of the most endangered species in the world and there are currently only ~1,600 surviving individuals [3]. It has been reported that germ cell-like cells can be differentiated *in vitro* from MSCs derived from mouse BM [4] and human umbilical cord [5, 6]. In addition to the organizational protective measures and assisted reproductive technologies currently underway, these MSC-derived germ cells may also be a potential resource useful for rescuing the species from extinction in the future [3]. Therefore an optimized platform for maintaining the proliferation of giant panda MSCs is very important for current giant panda protection strategies.

Members of the fibroblast growth factor (FGF) family play diverse roles in regulating cell proliferation, migration and differentiation during embryonic development [7–9]. Basic fibroblast growth factor (bFGF), a member of the FGF family, is often considered as a growth factor and differentiation inducer within the stem cell research field. Furthermore, bFGF has previously been demonstrated to play roles in maintaining MSCs differentiation potential and increasing their telomere length in various culture systems [10–14]. Moreover, bFGF has been shown to promote the proliferation of BM stromal stem cells and formation of cartilage and bone [15–17]. However, whether bFGF plays a role in promoting the proliferation of MSCs derived from giant panda BM has not been reported. In this study, we aimed to investigate the role of bFGF on the proliferation of giant panda BM-MSCs *in vitro*.

## Materials and Methods

### Culturing of mesenchymal stem cells

Mesenchymal stem cells were established by our research group [3]. The procedures of animal handling were reviewed and approved by the Ethical Committee of Qingdao Agricultural University (agreement No. 2013–16).

Mesenchymal stem cells were cultured at 37°C under 5% CO_2_ in complete medium supplemented with low-glucose Dulbecco’s modified Eagle medium (LG-DMEM; Hyclone, SH30021.01B, China), 10% fetal bovine serum (FBS; Gibco, 10099–141, USA), 10 ng/mL epidermal growth factor (EGF; R&D, 2028-EG-200, USA), 5 ng/mL bFGF (Peprotech, 100-18B, USA), 2 mM L-glutamine (Gibco, 35050–061, Japan), 1% penicillin streptomycin combination (Hyclone, SV30010, China). The adherent cells were cultured in 60 mm dishes (Corning, 430166, USA) at 37°C with 5% CO_2_ and digested with 0.25% trypsin-EDTA to passage in every three days.

### 3-(4,5-dimethyl-2-thiazolyl)-2,5-diphenyl-2-H-tetrazolium bromide (MTT) assay

For the MTT assay, cells were seeded into 96-well plates at low cell concentrations (10^3^ cells / well) and cultured for 48 hrs. After exposure with different concentrations of bFGF, medium was replaced with 50 μL MTT solution, which was produced by diluting MTT with LG-DMEM. After culturing for 4 hrs in the dark, 150 μL dimethylsulfoxide (DMSO) was added into each well. One hour later, the absorbance at 570 and 630 nm was measured using an enzyme-linked immunosorbent assay reader (Thermo Fisher, MK3, USA).

### 5-Bromo-2-deoxyUridine (BrdU) labeling and immunofluorescence

Initially, the MSCs were incubated in 30 μg/mL BrdU for 48 hrs. The cells were collected and fixed in 4% paraformaldehyde (Solarbio, P1110, China) for 15 min. After permeabilization with PBST which consist of 0.5% Triton X-100 (Solarbio, Amresco0694, China) and phosphate buffer solution (PBS), the cells were treated with 2 N HCl for 15 min. Blocking was performed in PBST containing 10% normal goat serum (Boster, AR0009, China) for 45 min and mouse anti-BrdU polyclonal antibody (1: 200, Sigma, B2531-2ML, USA) were incubated overnight at 4°C. Cells were then washed with PBS containing 1% bovine serum albumin (BSA; Solarbio, A8020, China), then the cells were incubated in 1: 50 Cy3-conjugated goat anti-mouse IgG (Beyotime, A0521, China) for 1 h at 37°C. Lastly, Hoechst 33342 (10 μg/mL, Beyotime, C1022, China) was used to stain the cell nuclei for 5 min. Immunofluorescence imaging was photographed using a laser scanning confocal microscope (Leica TCS SP5, Germany).

### Immunofluorescence staining

As described above, the MSCs were collected and fixed in 4% paraformaldehyde. After permeaibilization with PBST, the cells were blocked in PBST containing 10% normal goat serum for 45 min at room temperature and then goat anti-OCT4 polyclonal antibody (1: 200, Abcam, ab27985, HK) and were incubated overnight at 4°C. Then the cells were washed with PBS containing 1% BSA and incubated in 1: 50 Cy3-labeled Donkey anti-Goat IgG (Beyotime, A0502, China) at 37°C. Then PBS was used to remove redundant second antibody and the cell nuclei was stained with Hoechst 33342 for 5 min. With PBS washing again, immunofluorescence imaging was photographed.

### Cell cycle analysis

For cell cycle analysis, the number of MSCs at least needed to reach 10^6^. The cells were collected after digesting and fixed in an alcohol blend (70% alcohol and 30% PBS) at 4°C for at least 1 hr. After washed with PBS, the cells were incubated out of light in PBS containing 20 μg/mL propidium iodide (Abcam, ab14083, HK) and 1% RNaseA (Beyotime, ST578, China) at 37°C for 30 min. Then, before measurement, the cell samples were resuspended in PBS and analyzed with a FACS Calibur flow cytometry (Becton Dickinson, USA).

### Statistical analysis

All data were obtained from at least three replicates for each experiment. Data were presented as mean ± SEM. Differences among groups were statistically tested by Student’s t-test or one-way analysis of variance (ANOVA). Differences with P values less than 0.05 were considered significant.

## Results

### bFGF stimulates the proliferation of BM-MSCs *in vitro*

**Fig 1:**
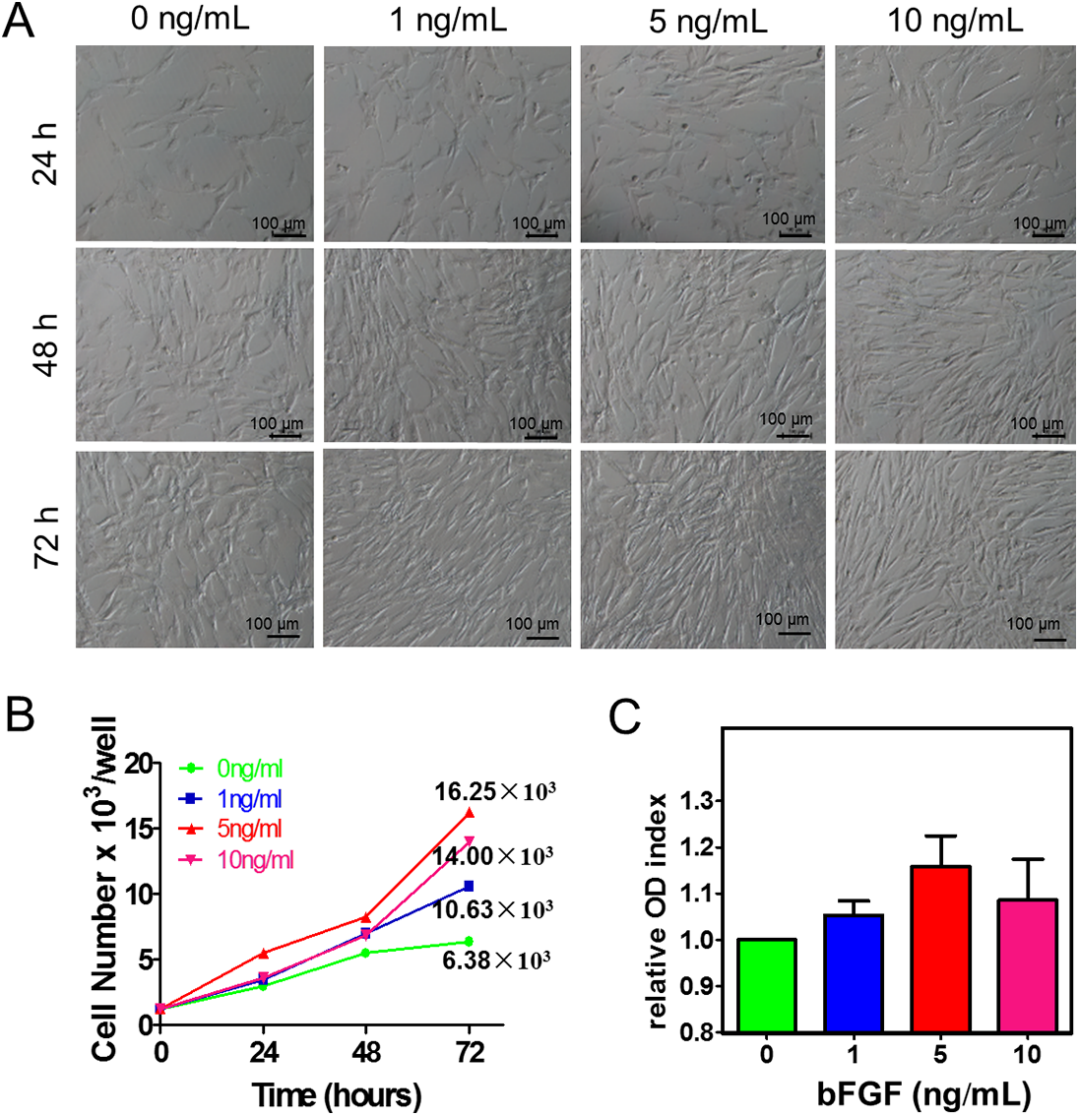
Detection of mesenchymal stem cell (MSC) proliferation. Morphology (A) and cell count (B) of panda bone marrow (BM) derived MSCs with different concentrations of basic fibroblast growth factor (bFGF) added at 24, 48 and 72 hrs of culture; (C) MTT proliferation assay of MSCs stimulated with 0, 1, 5 and 10 ng/ml of bFGF was evaluated at 48 hrs.

To investigate the role of bFGF on the proliferation of giant panda BM-MSCs, we seeded the passaged BM-MSCs into 24-well plates with complete medium supplemented with the different concentrations of bFGF (0, 1, 5 and 10 ng/mL) (Fig 1A). BM-MSCs were collected at 24, 48 and 72 hrs, following the addition of bFGF, for cell proliferation analysis. With increasing concentrations of bFGF, cell numbers of giant panda BM-MSCs gradually increased. This trend is indicated by cell counts at 72 hrs, the cell numbers of the 5 and 10 ng/mL groups were up to 2.55 and 2.20 times higher than that of the control group (Fig 1B; P < 0.05). Furthermore, the MTT data showed that, although there was no significant difference, the percentage of cells proliferating in the 5 ng/mL group was slightly higher than that of the other three groups (Fig 1C).

**Fig 2:**
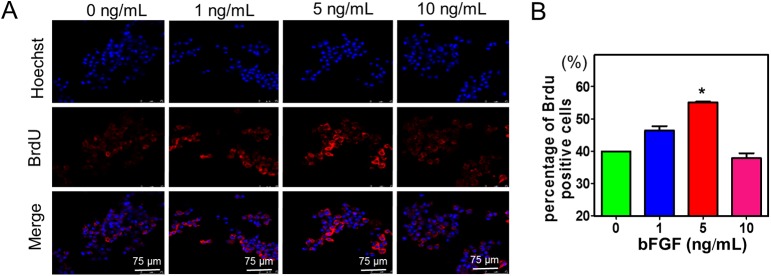
(A) BrdU labeling of MSCs supplemented by 0, 1, 5 and 10 ng/ml bFGF at 48 hrs. BrdU (red) dyes the proliferating cells. Hoechst 33342 (blue) stained the cell nuclei; (B) the percentages of BrdU positive cells in different concentrations of bFGF. * indicates significance of P < 0.05.

To further validate the proliferation effect of bFGF to BM-MSCs *in vitro*, immunofluorescence of BrdU labeled cells was carried out (Fig 2A). Results showed that the percentage of BrdU positive cells reached 55% in the 5 ng/mL group (Fig 2B), and it was significantly higher than that of control group (P < 0.05).

**Fig 3:**
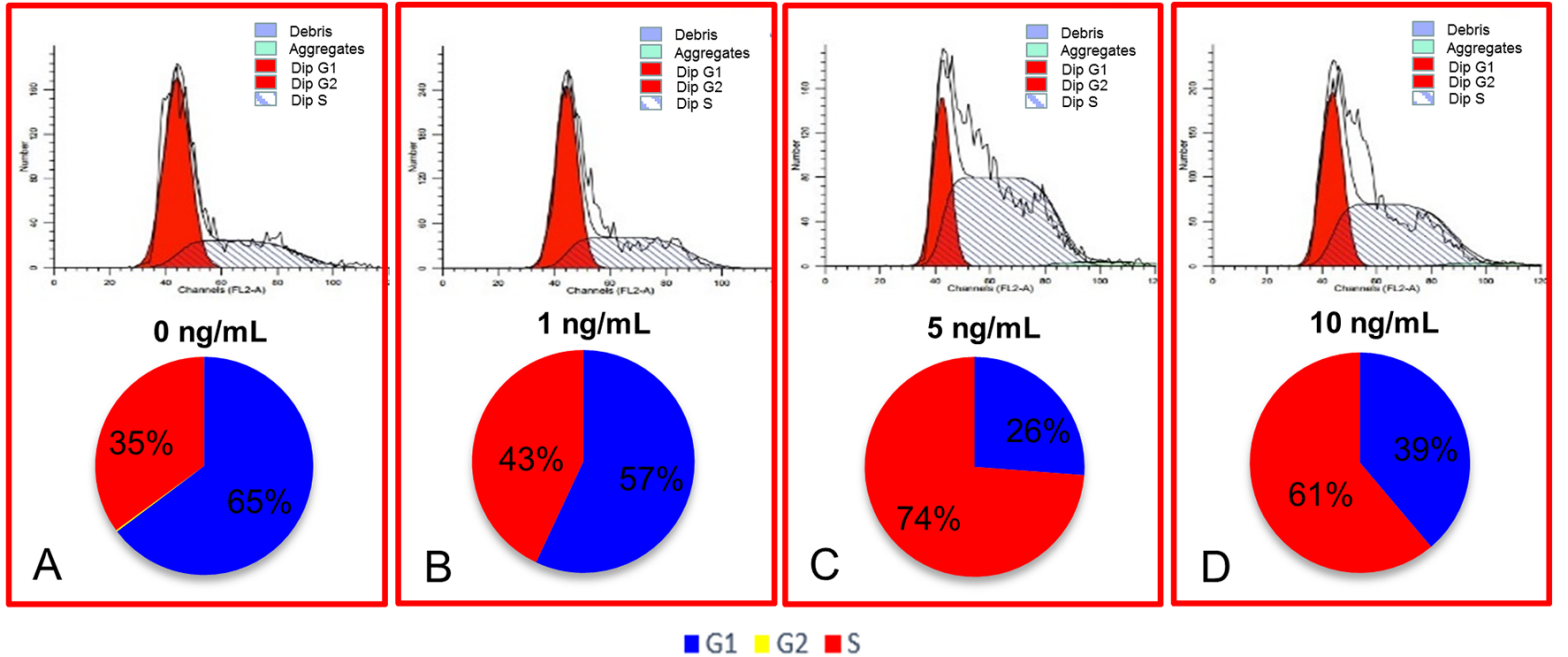
Cell cycle analysis of MSCs by FACS and the percentages of cells at different cell cycle phases in different concentrations of bFGF.

What's more, flow cytometry cell cycle analysis was utilized in our study. According to our data, the percentage of S phase cells in the 5 ng/mL group was 74%, which was more than double that of the control group (35%) (Fig 3; P < 0.05), and substantially higher than other two groups.

### bFGF affects the pluripotency of BM-MSCs *in vitro*

**Fig 4:**
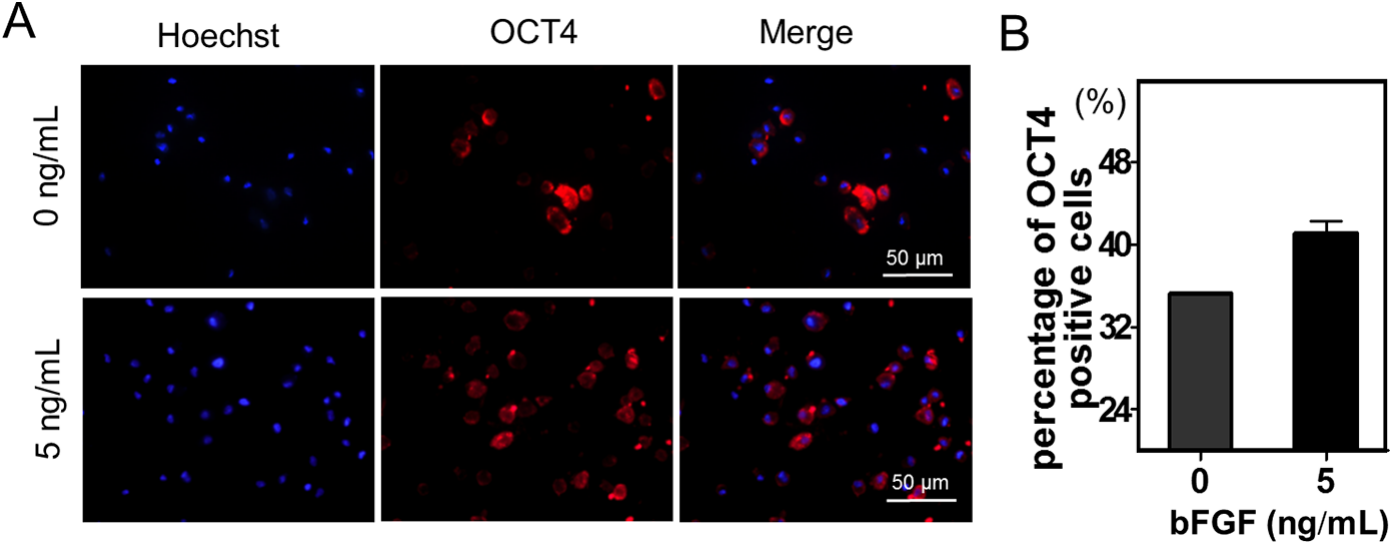
(A) OCT4 labeling of MSCs supplemented with 0, and 5 ng/ml of bFGF at 48 hrs. (B) The percentages of OCT4 positive cells cultured with 0 and 5 ng/ml of bFGF at 48 hrs.

The supplementation of stem cell culture medium with bFGF has been shown to enhance MSCs' trilineage differentiation capacity [18–20]. In order to investigate whether bFGF alters the pluripotency of the panda BM-MSCs, we collected cells that were cultured with or without bFGF after 48 hrs, and compared the expression of the pluripotency related protein OCT4. Results showed that the percentage of OCT4 positive cells increased slightly after treatment with 5 ng/mL bFGF (Fig 4A and 4B), meanwhile, the increasing percentage of OCT4 positive cells may result from the rising number of BM-MSCs.

### ERK signaling pathway plays a crucial role in the proliferation of BM-MSCs stimulated by bFGF

**Fig 5:**
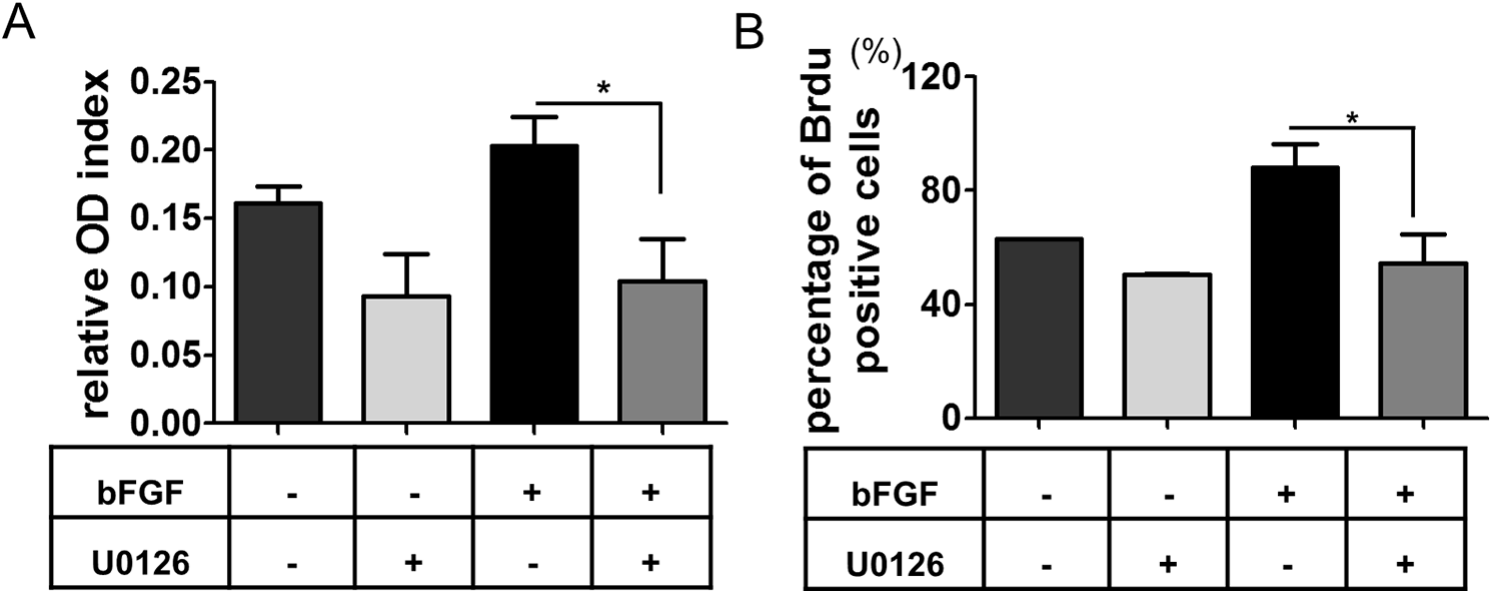
Detection of pluripotency and ERK signaling pathway on MSCs. (A) MTT assay of MSCs stimulated with 0 and 5 ng/ml of bFGF supplementation of U0126 at 48 hrs; (B) the percentages of BrdU positive cells supplementation with U0126 in the medium. * indicates significance of P < 0.05.

To explore whether the ERK signaling pathway plays a crucial role in the proliferation of panda BM-MSCs, U0126, the specific inhibitor of ERK, was added into the medium. MTT cell proliferation assay and BrdU labeling analysis were also performed. Our data displayed that the relative OD index in the bFGF treatment group with U0126 decreased significantly when compared to that of the bFGF treatment group without U0126 (Fig 5A; P < 0.05). Furthermore, the percentage of BrdU positive cells in the bFGF treatment group supplemented with U0126 were significantly lower than that of the bFGF treatment group without U0126 (Fig 5B and S1A Fig; P < 0.05). Thus, we suggested the ERK signaling pathway may play an important role in the proliferation of panda BM-MSCs stimulated by bFGF.

In order to study whether bFGF also inhibited cellular apoptosis in the process of promoting the proliferation of panda BM-MSCs, we performed cell apoptosis detection testing between the concentrations 0 and 5 ng/mL. The results suggest that bFGF is not involved in this process (S1B and S1C Fig).

## Discussion

In recent years MSCs have been investigated as promising candidates for use in new cell-based therapeutic strategies and genetic resource protection. They can be easily isolated from many adult tissues including bone marrow, adipose tissue, the umbilical cord, fetal liver, muscle, and lung [21–25]. They have the added benefit of relieving ethical concerns surrounding other sources of stem cells. For these reasons MSCs remain as the major stem cell type utilized for cell therapy, and have been used in the clinic for approximately 10 years [26]. Recently, several studies have indicated that MSCs derived from the BM could significantly impact wound healing in diabetic and non-diabetic animals [27]. Thus, MSCs have afforded promise in the treatment of numerous diseases, primarily tissue injury and immune disorders [26]. However, whether MSCs could be used for genetic resources protection of endangered animals has not been studied, particularly for the giant panda, one of the most widely recognized endangered species in the world. Here, we aimed to investigate the role of bFGF on the proliferation of BM-MSCs derived from the giant panda, which may provide valuable knowledge for future studies on possible stem cell applications in this endangered species. With the successful differentiation of stem cells into gamete-like cells, or even live progeny *in vitro*, MSC-derived germ cells may be a useful potential resource for rescuing the species from extinction in the future.

As an important mitogen, bFGF is a critical factor for maintaining the self-renewal process of human embryonic stem cells (hESCs) [28]. Although the bFGF treatment of MSCs will induce condrogenesis and osteogenic differentiation [29–31], there are still many reports suggesting bFGF supports the proliferation of MSCs [14, 32]. In addition, bFGF is one of the factors supporting the proliferation of preblastodermal cells [33], embryonic germ cells [34], and primordial germ cell (PGCs) [35] in chickens. Previous research has identified the optimal effect of bFGF on proliferation of dental pulp cells and apical papilla stem cells to be 100 ng/mL and 5 ng/mL respectively [36, 37]. In our research, an increase of bFGF concentration, resulted in an increase in the proliferation rate of giant panda MSCs up to 5 ng/mL. Our data here demonstrated that the cell proliferation rate in the 5 ng/mL bFGF group was higher than the other three groups, which was in accordance with a previous report that the cell proliferation rate of dog BM-MSCs showed a slight improvement when stimulated with 5 ng/mL bFGF for 24 hrs [32]. These reports and our data here suggest that an optimal concentration of bFGF on proliferative response must be determined in each individual cell line [38]. Our data also showed that the percentage of OCT4 positive cells increased slightly after treatment with 5 ng/mL bFGF, which may result from the rising number of BM-MSCs. Whether the pluripotency of the giant panda BM-MSCs *in vitro* was effected, there was not enough evidence to prove, because of limited specific antibodies against giant panda.

Furthermore, MEK/ERK is the most classical pathway in MAPKs and implicated in the regulation of numerous cellular processes including cell growth, differentiation and tumor progression. In 2008, Iván et al. revealed the functions of the ERK signaling pathway in human MSCs [18]. They found that the ablation of ERK2 gene expression (but not ERK1) by RNA interference (RNAi) significantly reduced the proliferation of hMSCs [18], which suggested that ERK2 may responsible for the proliferative enhancement ability of bFGF. Likewise, in mouse hepatocytes, ERK2 but not ERK1 is the key molecule involved in the induction of cell proliferation by bFGF [39]. Our data revealed that the ERK signaling pathway undoubtedly plays a crucial role in the proliferation of giant panda MSCs. Unfortunately, whether it's ERK2 that is activated by bFGF or essential for the proliferation of MSCs remains unclear.

In conclusion, this study suggests that giant panda BM-MSCs have a high proliferative capacity with 5 ng/mL bFGF *in vitro* and that the ERK signaling pathway is vital in this process. However, no specific antibodies against giant panda are commercially available at the present, our research was limited [3]. Whether ERK2 is responsible for the proliferation of panda BM-MSCs and the underlying molecular regulatory mechanism are still not well understood and thus need further study with specific antibodies against giant panda.

## Supporting Information

S1 Fig (A) BrdU labeling of MSCs supplemented with 0 and 5 ng/ml of bFGF at 48 hrs. BrdU (red) dyes the proliferating cells. Hoechst 33342 (blue) stained the cell nuclei; (B, C) Detection of apoptosis on MSCs with 0 and 5 ng/ml of bFGF. (TIF)
