# Supplementary figures and images for: Basic Fibroblast Growth Factor Stimulates the Proliferation of Bone Marrow Mesenchymal Stem Cells in Giant Panda (Ailuropoda melanoleuca)
Source: PLoS One. 2015 Sep 16;10(9):e0137712. doi: 10.1371/journal.pone.0137712 (PMC4574107; doi:10.1371/journal.pone.0137712)

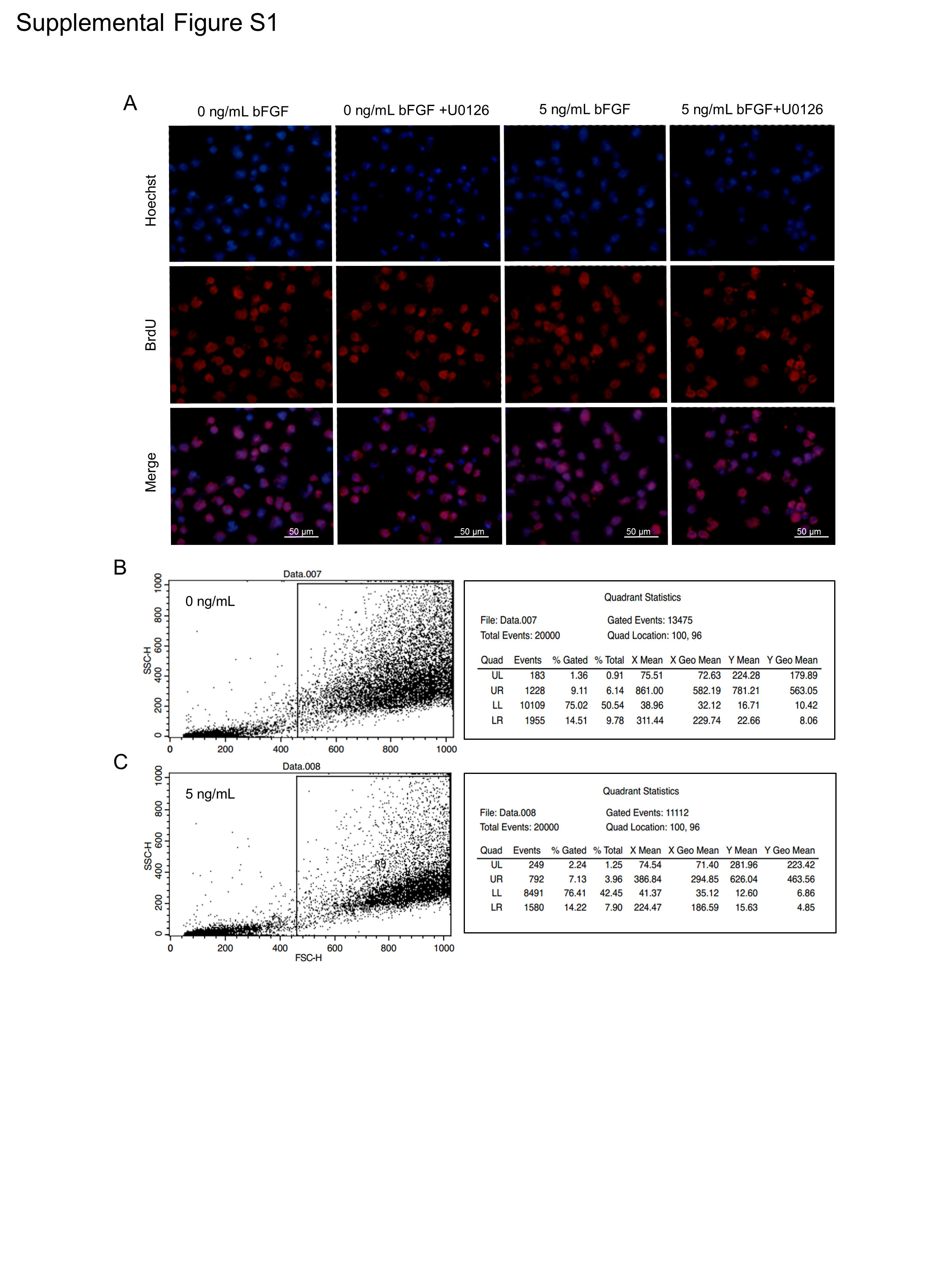

Supplement: S1 Fig — (TIF) [file pone.0137712.s001.TIF]
